# BRINGING COMMUNITY-BASED FALLS PREVENTION PROGRAM TO IMPROVE GLOBAL HEALTH (BRIGHT)

**DOI:** 10.1093/geroni/igad104.2561

**Published:** 2023-12-21

**Authors:** Ladda Thiamwong, Sirinart Tongsiri, Rui Xie, Boon Peng Ng, Joon-Hyuk Park, Wenjun Li, Elizabeth Eckstrom

**Affiliations:** University of Central Florida, Orlando, Florida, United States; Mahasarakham University, Mahasarakham, Maha Sarakham, Thailand; University of Central Florida, Orlando, Florida, United States; University of Central Florida, Orlando, Florida, United States; University of Central Florida, Orlando, Florida, United States; University of Massachusetts Lowell, Lowell, Massachusetts, United States; Oregon Health & Science University, Portland, Oregon, United States

## Abstract

Older adults in low- and middle-income countries (LMICs) experience a disparate burden of non-communicable diseases (NCDs) and barriers to accessing health care. Unintentional injuries are among the major NCDs, and falls are the second leading cause of these injuries and deaths. Falls were reported in up to 50% of older people living in Asia, including Thailand. There is a lack of community-based multifactorial fall prevention interventions that target both older adults and the primary care systems. We aimed to culturally adapt the CDC’s Stopping Elderly Accidents, Deaths, and Injuries (STEADI) for Thai older adults and explore the feasibility, appropriateness, and acceptability of using STEADI by trained community health workers (CHWs) and care managers (CMs). STEADI consists of three steps: screening, assessing, and intervening. In Step a, CHWs screened fall risk using three key questions and found that all of participants had fall risk, then CHWs screened with a Stay Independent questionnaire (range 0-14) and found that 100% have high fall risk (total scores 9.7± 2.4). In Step b, CMs assessed risk factors and CHWs assessed home hazards. They found that 50% had poor balance, 70% took 4+ medications, 75% fell on the walkway, and 70% had no bathroom modifications. In Step c, individual participants received fall prevention interventions and participants attended all sessions. Our results indicated that STEADI delivered by CHWs and CMs is feasible and acceptable for older adults. The mean scores of acceptability, appropriateness, and feasibility were 19.20,18.80, and 18.60 out of 20 totals, respectively.

